# Prognostic Value of β1 Adrenergic Receptor Autoantibody and Soluble Suppression of Tumorigenicity-2 in Patients With Acutely Decompensated Heart Failure

**DOI:** 10.3389/fcvm.2022.821553

**Published:** 2022-02-10

**Authors:** Yanxiang Sun, Li Feng, Bing Hu, Jianting Dong, Liting Zhang, Xuansheng Huang, Yong Yuan

**Affiliations:** Department of Cardiology, Zhongshan People's Hospital, Sun Yat-sen University, Zhongshan, China

**Keywords:** β1 adrenergic receptor (β1-AR), autoantibodies, heart failure, soluble suppression of tumorigenicity-2 (sST2), mortality

## Abstract

**Background:**

Both β1 adrenergic receptor autoantibody (β1-AA) and soluble suppression of tumorigenicity-2 (sST2) take a role in the pathological remodeling of heart failure. However, limited studies investigated the correlation between the expression of β1-AA and sST2 in patients with acutely decompensated heart failure (ADHF).

**Objective:**

To explore the correlation between β1-AA and sST2, and evaluate their prognostic value in patients with ADHF.

**Methods:**

Patients who were admitted for ADHF were included. The N-terminal pro-brain natriuretic peptide (NT-proBNP), sST2, and β1-AA in blood samples were tested at hospital admission and then followed up for assessing the outcomes. Pearson correlation analysis was used to explore the correlation between β1-AA and sST2. The effects of β1-AA, sST2, or the combination of them on the all-cause mortality of patients with ADHF were assessed by Multivariate Cox regression analysis.

**Results:**

There were 96 patients with ADHF and 96 control populations enrolled. The β1-AA was significantly higher in ADHF than in the control group (0.321 ± 0.06 vs. 0.229 ± 0.04, *P* = 0.000). Pearson correlation analysis showed that β1-AA was positively correlated with sST2 (*r* = 0.593), NT-proBNP (*r* = 0.557), Procalcitonin (*r* = 0.176), and left ventricular end-diastolic diameter (*r* = 0.315), but negatively correlated with triglycerides (*r* = −0.323), and left ventricular ejection fraction (*r* = −0.430) (all *P* < 0.05) in ADHF. Patients with ADHF, complicated with both high β1-AA and sST2, showed the highest all-cause mortality during an average of 25.5 months of follow-up. Multivariate Cox regression showed the combination of both high β1-AA and sST2 independently correlated with the all-cause mortality after adjustment for other risk factors (hazard ratio 3.348, 95% CI 1.440 to 7.784, *P* = 0.005). After adding with β1-AA and sST2, the area under the curves for the prognostic all-cause mortality could increase from 0.642 to 0.748 (*P* = 0.011).

**Conclusion:**

The β1-AA is positively correlated with sST2 in patients with ADHF. Elevated plasma β1-AA and sST2 level in patients with ADHF are associated with poorer prognoses.

## Introduction

Heart failure (HF) is a terminal stage of heart disease characterized by high mortality, which becomes more and more prevalent and brings a huge burden to health care (1). Acutely decompensated HF (ADHF) may have a higher in-hospital mortality risk (2). On the other hand, HF is associated with various pathophysiological and biochemical disorders. No single biomarker can display all these characteristics. Therefore, earlier identification of patients with ADHF, with high mortality risk and more sensitive assessing prognosis by the combination of cardiac multi markers that implement effective cardiovascular preventive measures, can improve the survival of those patients.

The β1 adrenergic receptor (β1-AR) is a G protein-coupled receptor that triggers physiological or pathological responses *via* activating the signaling cascade of adenylate cyclase, cyclic adenosine 3',5'-monophosphate, and protein kinase A. This signaling pathway regulates intracellular calcium concentration and determines cardiomyocyte contractility (3). The β1 adrenergic receptor autoantibody (β 1-AA) is a kind of autoantibody existing in the serum of patients with heart failure (4), which binds to the second extracellular loop of the β1-AR. The β1-AA is found in 30–75% of patients with dilated cardiomyopathy (DCM) (5, 6), which appear to be functionally active and are associated with a markedly worse prognosis in DCM (7). The β 1-AA is characterized by continuous activation without desensitization in HF, which result in intracellular calcium overload and myocardial apoptosis, promoting cardiac remodeling, prolonging QT dispersion to malignant arrhythmias, and cardiac sudden death (5, 8). Studies on clinical heart failure found that the β 1-AA can be antagonized by β-blockers and can be eliminated through immunoadsorption, which reverses the pathological remodeling and improves cardiac function (9, 10).

Soluble suppression of tumorigenicity-2 (sST2) has been implicated in the pathogenesis of myocardial fibrosis and remodeling in HF (11). The sST2 concentration is not influenced by age, kidney function, or body mass index, unlike the natriuretic peptides (12). So, the sST2 provides additional prognostic value over N-terminal pro-brain natriuretic peptide (NT-proBNP) in the prediction of death in patients with ADHF (13). At present, the sST2 assay has been tested for diagnosis and prediction in either acute or chronic HF populations (13, 14).

The involvement of both β1-AA and sST2 in the pathological remodeling of HF suggests a relationship between them. However, few studies investigated the correlation between the expression of β1-AA and sST2 in patients with ADHF. The current study aimed to analyze the correlation between the β1-AA and sST2. Furthermore, we also investigated whether the combination of β1-AA and sST2 was more accurate than β1-AA or sST2 alone in predicting the short-term survival in patients with ADHF.

## Materials and Methods

### Study Population

The study was approved by the Medical Ethics Committee of the Zhongshan People's Hospital, Guandong Province, China. All the participants in this study had signed written informed consents.

According to the 2021 European Society of Cardiology (ESC) Guidelines for the diagnosis and treatment of acute and chronic heart failure (2), the patients included in the study should have the signs and symptoms which indicated they had an attack of new-onset HF or acutely decompensated chronic HF (e.g., dyspnea, edema, weight gain). Also, they should be observed with elevated levels of NT-proBNP and the impaired systolic or diastolic function of the heart by echocardiography. The control populations without heart failure were from the physical examination center or outpatient clinic of Zhongshan People's Hospital. The exclusion criteria were age <18 years, acute coronary syndrome, unstable hemodynamics, cardiac surgery, malignant tumor, serious hepatic dysfunction (ALT ≥3ULN), kidney insufficiency (eGFR <60 ml/min·1.73m^2^ or Scr ≥ 265 umol/L), and cerebrovascular diseases. Between November 2018 and October 2019, 228 patients with ADHF were enrolled prospectively. At last, 96 patients with ADHF were enrolled because of deaths during hospitalization, loss to follow-up, and missing data. Ninety-six control populations without heart failure were selected for baseline comparison.

### Baseline Data Collection

Clinical data of each patient would be collected including age, gender, hypertension and diabetes history, and smoking. Furthermore, the results of laboratory examination such as Serum Creatinine (Scr), Total cholesterol (TC), Low-Density Lipoprotein Cholesterol (LDL-c), triglycerides (TG), C-reactive protein (CRP), and Procalcitonin (PCT) would be recorded. Left ventricular ejection fraction (LVEF) and left ventricular end-diastolic diameter (LVEDd) were tested with Simpson's method. Blood samples for the tests were obtained from each participant within the first 24 h after admission. We centrifuged blood samples at 18,000 g for 10 min and stored the serum samples at −80°C.

### Follow-Up

The follow-up period would start from the first day when the participants were discharged. The subjects would be investigated by the cardiologists. All the patients with ADHF were treated with standard HF management according to the current clinical practice guidelines, the control group were treated with antihypertensive or antidiabetic drugs if needed. Data of an all-cause mortality was collected with the means of regular telephone interviews, administrative databases, and medical records monthly.

### Biochemical Measurement

The serum sample of β1-AA was sent to the Laboratory of Cardiovascular Immunology, Union Hospital, Tongji Medical College of Huazhong University of Science and Technology. The level of β1-AA was measured by the enzyme linked immunosorbent assay (ELISA) as previously described (15). The optical density (OD) was determined using an ELISA plate reader at 450 nm. Serum sST2 was measured with a human ST2 Quantikine ELISA Kit (R&D Systems, Inc., Minneapolis, Minnesota, United States). Serum NT-proBNP was tested by electrochemiluminescence immunoassay (Roche, Basel, Switzerland).

### Statistical Analysis

Data were expressed as mean ± SEM or median (interquartile range, IQR). Comparison of continuous variables between two groups was performed by analysis of variance with the independent-sample student's *t*-test. Otherwise, the rank-sum test was used. Categorical data such as the incidence of disease were analyzed by chi-square test. Pearson correlation analysis was used for correlation analysis between β1-AA and sST2. Kaplan-Meier assessed the effect of different levels of β1-AA and sST2 (above or below mean) on the survival rate of patients with heart failure by Log-rank test. Cox regression analysis and LR Forward (*P* < 0.05 in, >0.1 out) was performed to identify the risk factors for an all-cause mortality. To evaluate the improvement in predictive accuracy, we built the receiver operating characteristic (ROC) curves of the risk prognostic models and calculated the area under the curves (AUC), respectively. The comparisons between models by a pair-wise method were performed with the method of Hanley JA and McNeil BJ (16). The IBM SPSS Statistics 26.0 software was used for statistical analysis (IBM SPSS Inc., Chicago, USA). Values of *P* < 0.05 were considered to denote statistical significance.

## Results

### Patient Characteristics

The baseline characteristics of patients with ADHF and the control population enrolled were shown in Table 1. There was no significant difference in age, gender, LDL level, hypertension, and smoking history between the two groups, but the incidence of diabetes (*P* = 0.018) and median follow-up of an all-cause death (*P* < 0.01) were significantly higher in the ADHF group than in the control group. The TC and TG in the ADHF group (4.11 ± 1.14 mmol/L and 1.16 ± 0.53 mmol/L) were lower than the control group (4.58 ± 0.98 mmol/L and 1.70±0.62 mmol/L), but the mean concentration of NT-proBNP and sST2 is 5543 (IQR, 2,889.75, 10,667.25) pg/ml and 21.21 (IQR, 12.89, 41.26) ng/ml in the ADHF group, which were significantly higher than those in the control group. Left ventricular ejection fraction (LVEF) in the ADHF group (46.15%±10.43%) was lower than that in the control group (65.53%±6.11%), while LVEDD was significantly higher in the ADHF group (53.85 ± 10.49 mm) than that in the control group (46.02 ± 4.83 mm). The OD value of β 1-AA in the ADHF group (0.321±0.06) was also significantly higher than that in the control group (0.229±0.04). The levels of PCT, serum creatinine, and C-reactive protein in the ADHF group were higher than those in the control group (all *P*-values < 0.01).

**Table 1:** Baseline characteristics of patients.

	**ADHF (*N =* 96)**	**Control (*N =* 96)**	***P***
Male, *n* (%)	54 (56.5%)	53 (55.2%)	0.500
Age (years)	71.53 ± 14.24	70.69 ± 12.57	0.668
Hypertension, *n* (%)	59 (61.6%)	54 (56.3%)	0.279
Diabetes, *n* (%)	23 (23.9%)	11 (11.5%)	0.018
Mortality, *n* (%)	39 (40.6%)	0 (0)	0.000
Smoking, *n* (%)	39 (40.6%)	31 (32.3%)	0.147
NT-proBNP (pg/mL) ^Δ^	5,543	93.80	0.000
	(2,889.75, 10,667.25)	(27.25,187.50)	
sST2 (ng/mL) ^Δ^	21.21 (12.89,41.26)	8.49 (4.79, 13.81)	0.000
β1-AA OD	0.321 ± 0.06	0.229 ± 0.04	0.000
LVEF%	46.15 ± 10.43	65.53 ± 6.11	0.000
LVEDd (mm)	53.85 ± 10.49	46.02 ± 4.83	0.000
TC (mmol/L)	4.11 ± 1.14	4.58 ± 0.98	0.000
TG (mmol/L)	1.16 ± 0.53	1.7 ± 0.62	0.000
LDL-c (mmol/L)	2.53 ± 0.97	2.55 ± 0.74	0.872
PCT (ng/mL) ^Δ^	0.064 (0.035, 0.133)	0.035 (0.022,0.049)	0.000
SCr (umol/L) ^Δ^	107.00 (83.25, 125.75)	74.00 (62.00,90.25)	0.000
CRP (mg/L) ^Δ^	10.80 (2.84, 20.30)	1.50 (0.70,3.50)	0.000

*Data are presented as the x± SD, the median (^Δ^); sST2, soluble suppression of tumorigenicity-2; β1-AA, β1-adrenoceptor autoantibody; TC, Total cholesterol; TG, triglycerides; LDL-c, low-density lipoprotein cholesterol; LVEF, left ventricular ejection fraction; LVEDd, left ventricular end-diastolic diameter; NT-proBNP, N-terminal proB-type natriuretic peptide; PCT, procalcitonin; and SCr, serum creatinine*.

*P values are comparison between ADHF and Control groups*.

### Correlation Analysis Between β 1-AA and Other Indicators of Heart Failure

Age, sST2, NT-proBNP, CRP, PCT, CR, TC, TG, LVEF, and LVEDD values were included in the Pearson correlation analysis for all data. Results showed that sST2 (*r* = 0.525, *P* < 0.01) and TG (*r* = −0.305, *P* < 0.01) were correlated with the value of β1-AA in all population. In the ADHF group, sST2 (*r* = 0.593, *P* < 0.01), NT-proBNP (*r* = 0.557, P < 0.01), PCT (*r* = 0.176, *P* = 0.026), LVEDd (*r* = 0.315, *P* < 0.01), TG (β = −0.323, *P* < 0.01), and LVEF (β = −0.430, *P* < 0.01) were correlated with the β1-AA level (Table 2).

**Table 2:** Correlation between the β1-AA and indexes by Pearson correlation analysis in all population and patients with ADHF.

	**All population**		**ADHF**	
	***r***	***P***	***r***	***P***
sST2	0.525	0.000	0.593	0.000
NT-proBNP	0.189	0.066	0.557	0.000
TG	−0.305	0.005	−0.323	0.000
*PCT*	0.105	0.329	0.176	0.026
LVEDd	0.088	0.392	0.315	0.000
LVEF	−0.183	0.074	−0.430	0.000

*2Log transformations were made for the sST2, NT-proBNP, and PCT in the analysis because of their skewed distribution*.

### Effects of Different Levels of the β1-AA and SST2 on the All-Cause Mortality in Patients With ADHF

Groups were divided according to the mean of β1-AA and the median of sST2 in the patients with ADHF: the high-value group and the low-value group. Cumulative all-cause mortality was higher in the high β1-AA or sST2 groups than in the low β1-AA or sST2 groups (respectively, 25 vs.15.63%, *P* = 0.017; 26.04 vs. 14.58%, *P* = 0.014). After the combination of β1-AA and sST2, the cumulative all-cause mortality of both high-value groups was significantly higher than that of the low-value groups (27.14 vs. 12.85%, *P* = 0.018) by chi-square test (Table 3).

**Table 3:** The relationship between levels of the β1-AA, sST2 and the all-cause mortality in patients with ADHF by Chi-square analysis.

	**All-cause mortality % (n)**		***chi-square value***	***df***	***P-value***
	**High**	**Low**			
β1-AA (*N =* 96)	25% (24)	15.63% (15)	5.671	1	0.017
sST2 (*N =* 96)	26.04% (25)	14.58% (14)	6.028	1	0.014
β1-AA+sST2 (*N =* 70)	27.14% (19)	12.85% (9)	8.037	1	0.018

*High: the level of β1-AA was above the mean in β1-AA line, the level of sST2 was above the median in sST2 line, the level of β1-AA and sST2 was both above their mean or median in β1-AA+ sST2 line*.

*Low: the level of β1-AA was below the mean in β1-AA line, the level of sST2 was below the median in sST2 line, the level of β1-AA and sST2 was both below their mean or median in β1-AA+ sST2 line*.

*Compared with the low value group, the all-cause mortality in the high value group was significantly increased, and P < 0.05*.

### The β1-AA and SST2 Are Independent Risk Factors for an All-Cause Death in Patients With ADHF

**Figure 1:**
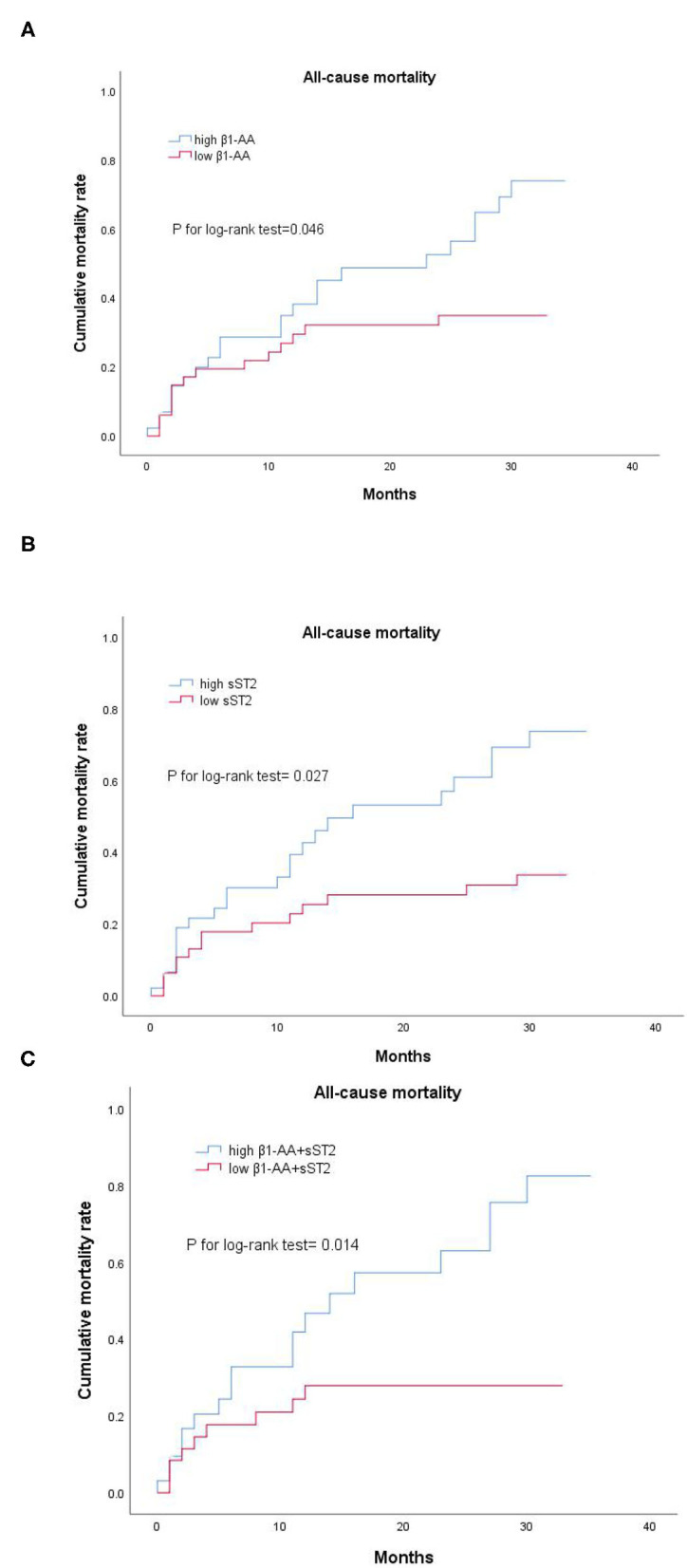
**(A–C)** Survival analysis of β1-AA and sST2 baseline levels in patients with ADHF. Kaplan-Meier analysis showed that patients with high β1-AA and sST2 had obviously higher cumulative rates of the all-cause mortality than those with low β1-AA and sST2. High β1-AA and high sST2, the level of β1-AA and sST2 was above their mean or median; low β1-AA and low sST2, the level of β1-AA and sST2 was below their mean or median. High β1-AA+ sST2, the level of β1-AA and sST2 was both above their mean or median; low β1-AA+ sST2, the level of β1-AA and sST2 was both below their mean or median.

A total of 228 patients were discharged, except for 32 deaths during hospitalization. Thirty-six other cases were lost with follow-up information and 64 data were lacking during an average of 25.5 ± 1.47 months of follow-up. There was no incidence of death in the control group during follow-up, we, thus, analyzed the effect of the β1-AA and sST2 on the prognosis only in patients with ADHF. We group according to the mean levels of β1-AA and median levels of sST2. Kaplan-Meier analysis showed that patients with high β1-AA, high sST2, and both high β1-AA and sST2 showed a higher cumulative rate of an all-cause mortality than those with low β1-AA, low sST2 and both low β1-AA and sST2 (*P* for log-rank test =0.046,0.027, and.014, respectively, Figures 1A–C). Multivariate Cox regression analysis showed the high β1-AA (*HR* 2.199; 95% *CI* 1.106–4.373, *P* = 0.025), high sST2(*HR* 2.333*;* 95% *CI* 1.173–4.638, *P* = 0.016), and the combination of both high β1-AA and sST2 (*HR* 3.348; 95% *CI* 1.440–7.784, *P* =0.005) were independent risk factors for all-cause mortality after adjusting other risk factors (Table 4).

**Table 4:** Association between level of β1-AA/sST2 and all-cause mortality by Multivariate Cox regression.

	**B**	**S.E**.	**W value**	**HR**	**95 % CI**	***P***
β1-AA^#^	0.788	0.351	5.05	2.199	1.106–4.373	0.025
sST2^*^	0.847	0.351	5.381	2.333	1.173–4.638	0.016
β1-AA+ sST2^§^	1.208	0.430	7.880	3.348	1.440–7.784	0.005

*β1-AA, sST2 and combination of β1-AA and sST2 were independent risk factors for an all-cause mortality in ADHF by multivariate cox regression, all P < 0.05. B, regression coefficient; S.E., standard error; W value, Wald chi-square value; HR, hazard ratio; CI, confidence interval*.

# *adjust for age, diabetes, sST2, NT-proBNP,and PCT*.

* *adjust for age, diabetes, β1-AA, NT-proBNP, and PCT*.

§ *adjust for age,diabetes, NT-proBNP, and PCT*.

### The Combination of β1-AA and SST2 Provides Incremental Prognostic Value in the Survival of Patients With ADHF

Model 1 consisted of age, diabetes, NT-proBNP, and PCT. Model 2 was made up of Model 1 and β1-AA. Model 3 was constructed by Model 1 with Log2 sST2. Model 4 was built by combining Model 1 with β1-AA and Log2 sST2. Table 5 showed the ROC curves of models for predicting the all-cause mortality of patients with ADHF, in which the AUC of Models 1, 2, 3, and 4 were 0.642 (95% CI.519–0.765),0.714 (95% CI.599–0.830), 0.735 (95% CI.625–0.846), and.748 (95% CI.638–0.858), respectively (all *P* < 0.05). Among them, the area under the curves (AUC) of Model 2, 3 and 4 performed better than that of Model 1 (respectively, ΔAUC.072, *P* = 0.041; ΔAUC 0.093, *P* = 0.018; ΔAUC 0.106, *P* = 0.011), but the prognosis of all-cause mortality had no significant difference among Models 2, 3, and 4 (AUC4-AUC2 = 0.034, AUC4-AUC3 = 0.013, both *P* > 0.05). More details about the different ROC curves of models were shown in Table 5. The result showed that the risk stratification value of β1-AA and sST2 was additive in patients with ADHF.

**Table 5:** The AUC and Δ AUC of the ROC curves from different models and the comparisons.

	**AUC**	**95% CI**	***P***	**ΔAUC**	**P^Δ^**
Model 1	0.642	0.519–0.765	0.024		
Model 2	0.714	0.599–0.830	0.001	0.072^#^	0.041
Model 3	0.735	0.625–0.846	0.000	0.093^*^	0.018
Model 4	0.748	0.638–0.858	0.000	0.106^§^	0.011

*Model 1: age, diabetes, NT-proBNP and PCT; Model 2: Mode l +β1-AA; Model 3: Mode l + Log 2 sST2; Model 4: Mode l +β1-AA+ Log 2 sST2*.

# *ΔAUC, Model 2-Model1;*

* *ΔAUC, Model 3-Model1;*

§ *ΔAUC, Model 4-Model1*.

*AUC, the area under the curves. ΔAUC, the difference of two AUC. P, the significance of the probability for ROC curves of models for predicting the all-cause mortality of patients with ADHF. P^Δ^, the significance of the probability for difference of ΔAUC. CI, confidence interval*.

## Discussion

A number of studies have suggested that the β1-AA has an important role in pathophysiological processes of heart failure (6, 9, 10, 17) The current study found that the β1-AA was positively correlated with sST2 and NT-proBNP in ADHF. Therefore, this study demonstrates for the first time the internal relationship between the expression of β1-AA and sST2. It has been suggested that the inflammatory factors, including interleukine-6 (IL-6), various viral and microbial candidate proteins can promote β1AR-directed autoimmune cardiomyopathy through enhancing the β1-AA production (15, 18, 19). The sST2 is also involved in inflammatory diseases. The sST2 is increased in conditions of cardiac and vascular stress, particularly in HF. (14, 20) Interestingly, sST2 induces the secretion of IL-6, a pro-inflammatory cytokine that stimulates β1-AA production, leading to a cardiac damage by promoting oxidative stress and inflammation (21). Therefore, we speculate that the high-level sST2 in patients with ADHF promotes the secretion of IL-6, which increases the production of β1-AA and exaggerates myocardial injury. This provides a possible explanation for the positive correlation between the β1-AA and the sST2. Similar to the effect of sST2, the β1-AA can also induce an IL-6 secretion through mediating T lymphocyte disorder, aggravating cardiac remodeling (17). In addition, β1-AA and the sST2 activate a similar intracellular signal pathway that induces myocardial fibrosis. Studies showed that the β1-AA promotes proliferation in cardiac fibroblasts through activating p38 mitogen-activated protein kinase (p38MAPK) through specifically binding to the β1-AR (7), leading to a myocardial fibrosis. The sST2 can also activate p38MAPK and nuclear factor-κB (NF-κB) signaling pathways, inducing ventricular remodeling, and fibrosis (11, 22). Of note, both β1-AA and the sST2 in promoting intracellular p38MAPK phosphorylation can be antagonized by β blockers (10, 11). Studies have shown that the dose of β-blockers in patients with HF may affect the concentration of sST2, and the combination of sST2 value and the dose of beta-blockers can be used for risk stratification on the prognosis of patients with HF (23). Therefore, the positive correlation between β1-AA and sST2 may be that sST2 promote the secretion of β1-AA on the one hand, and on the other hand, both of them share a common intracellular signaling pathway that causes myocardial fibrosis and is antagonized by β blockers, so, they are both interrelated and synergistic in inducing myocardial fibrosis. Consequently, we speculated that the degree of myocardial fibrosis may be more obvious when the β1-AA and sST2 are elevated simultaneously in patients with heart failure. So far, there is no relevant report and the next study of synergistic effect of β1-AA and sST2 on promoting cardiac fibrosis will be testified by cardiac MRI in our future study.

Another important finding in this study is that the β1-AA and sST2 were, respectively, independent risk factors for a short-term all-cause death in patients with ADHF, and higher levels of these biomarkers were associated with a higher risk of an all-cause death. Of particular significance, the combination of β1-AA and sST2 could improve risk stratification in predicting short-term survival among patients with ADHF. An animal study confirmed that the NT-proBNP was increased and the left ventricular shortening rate was decreased in rats immunized with β1-AA (18). Meanwhile, the β1-AA level is an independent predictor of myocardial reverse remodeling (24). The β1-AA reduces cardiac function, enhances arrhythmia, and sudden cardiac death, (5, 8, 25) so it can be used as a predictor for death risk in patients with heart failure (8, 26). Consistent with previous findings, this study showed that the β1-AA was positively correlated with NT-proBNP and negatively correlated with the EF value in ADHF. The current results indicated that β1-AA could accelerate myocardial remodeling and was an independent risk factor for a short-term all-cause death in patients with ADHF.

Many novel biomarkers had been explored in the prognosis of HF, such as Secreted frizzled-related proteins (Sfrps) (27–29) and Trimethylamine N-oxide (TMAO) (30). In addition, our previous study demonstrated the biomarkers of myocardial fibrosis, such as sST2 and Procollagen Type III N-Terminal Peptide (PIIINP), are independent prognostic factors for all-cause mortality in patients with ADHF (31), and the combination of biomarkers could be more sensitive to predict an all-cause death in patients with ADHF. Our finding showed that the β1-AA and sST2 were, respectively, independent predictors for short-term all-cause death in patients with ADHF after adjustment for other risk factors. Importantly, the combination of β1-AA and sST2 is a stronger predictive for an all-cause mortality in patients with ADHF.

## Limitations

Limitations of the presented study include that this study was performed on a single center with small sample size and many confounders cannot be adjusted, the conclusion of which still warrants confirmation in multiple centers with larger sample sizes. In addition, the current study only analyzed the levels of the β1-AA and sST2 in patients when they were admitted to the hospital. We should take the blood sample after the HF guideline-directed management at different periods. It is more helpful to elucidate the relationship between the expression of the biomarkers and the prognosis of ADHF.

Further studies should establish the optimal timing of β1-AA and sST2 sampling, and more importantly, assess whether specific action in response to the prognostic information conveyed by β1-AA and sST2 indeed improves patient status and outcomes.

## Conclusions

The current study confirmed that the β1-AA was positively correlated with sST2 in patients with HF. Furthermore, elevated plasma β1-AA and sST2 level in patients with patients is associated with poorer prognoses.

## Data Availability Statement

The raw data supporting the conclusions of this article will be made available by the authors, without undue reservation.

## Ethics Statement

Written informed consent was obtained from the individual(s) for the publication of any potentially identifiable images or data included in this article.

## Author Contributions

YS wrote and edited the manuscript. YS, BH, LF, JD, and LZ collected the research data for the article. XH and YY reviewed the manuscript and approved the final manuscript. All authors contributed to the article and approved the submitted version.

## Funding

This work was supported by Medical Scientific Research Foundation of Guangdong Province, China (No. A2013864).

## Conflict of Interest

The authors declare that the research was conducted in the absence of any commercial or financial relationships that could be construed as a potential conflict of interest.

## Publisher's Note

All claims expressed in this article are solely those of the authors and do not necessarily represent those of their affiliated organizations, or those of the publisher, the editors and the reviewers. Any product that may be evaluated in this article, or claim that may be made by its manufacturer, is not guaranteed or endorsed by the publisher.
